# Supplementary material for: Plant Growth Promotion and Biological Control against Rhizoctonia solani in Thai Local Rice Variety “Chor Khing” Using Trichoderma breve Z2-03
Source: J Fungi (Basel). 2024 Jun 11;10(6):417. doi: 10.3390/jof10060417 (PMC11204415; doi:10.3390/jof10060417)
Supplement: Supplementary file 1 [file jof-10-00417-s001.zip › jof-3011167-supplementary.pdf]

Plant Growth Promotion and Biological Control Against *Rhizoctonia solani* in Thai Local Rice Variety "Chor Khing" using *Trichoderma breve* Z2-03

The experiments consisted of: i) effect of cell free-CF on seed germination, ii) increase in rice seedling growth caused by *Trichoderma* isolate Z2-03, iii) total chlorophyll content, iv) antifungal ability of *Trichoderma* sp. Z2-03, v) siderophore production of *Trichoderma* sp. *Z2-03* and vi) *Trichoderma* promote plant growth and reduce disease incidence were repeated twice. This supplementary file demonstrates the results of both experiments.

1. Effect of Cell Free-CF on Seed Germination

The effect of *Trichoderma* isolate Z2-03 cell-free CF on the induction of plant growth was measured on germinated rice at 4 day post application (dpa). The application of the selected *Trichoderma* isolate Z2-03 cell-free CF led to root and shoot lengths that were significantly greater than those in the control group (Fig. 1S). At 4 dpa, the shoot lengths were 1.14 ± 0.14, 1.15 ± 0.18 and 1.98 ± 0.33 cm, respectively, whereas the root lengths of the DW, PDB and cell-free CF-treated seeds were 2.83 ± 0.48, 2.69 ± 0.43 and 3.84 ± 0.35 cm, respectively ($p < 0.05$). At 5 dpa, both the root and the shoot showed continuous germination; the shoot lengths of DW, PDB and cell-free CF-treated seeds were 1.51 ± 0.19, 1.40 ± 0.24 and 2.46 ± 0.45 cm, respectively, whereas the root lengths of the DW, PDB and cell-free CF-treated seeds were 2.90 ± 0.49, 2.77 ± 0.40 and 3.91 ± 0.43 cm, respectively ($p < 0.05$). The results of the second experiment also indicated that treatment with *Trichoderma* sp. Z2-03 cell free-CF resulted in the highest shoot and root length of germinating seeds (Fig. 2S).

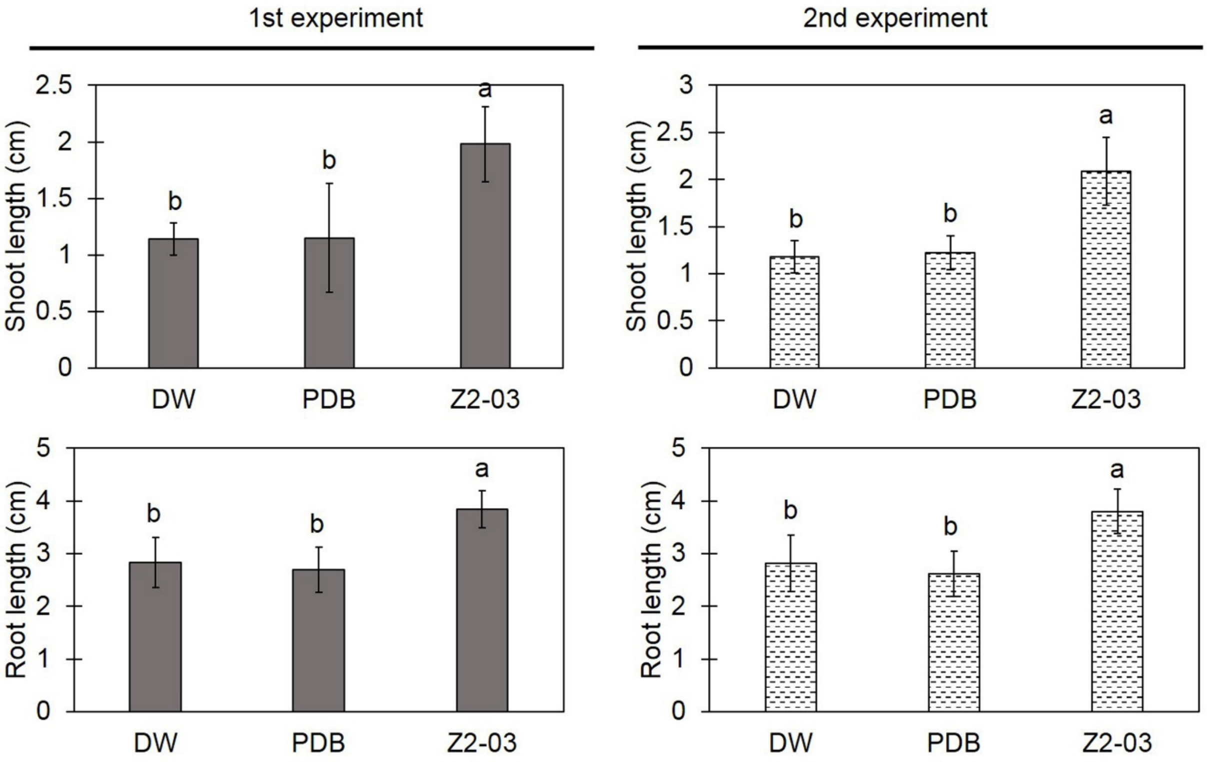

Figure S1. Seed germination, as well as shoot and root length, of "Chor Khing" rice variety assessed using distilled water (DW), potato dextrose agar broth (PDB) and the cell-free culture filtrate of *Trichoderma* isolate Z2-03 (Z2-03) from 1st and 2nd experiment at 4 day post application (dpa). Values are shown as mean ± SD, with letters indicating significant differences among the control and the treatment according to Tukey's test ($p < 0.05$).

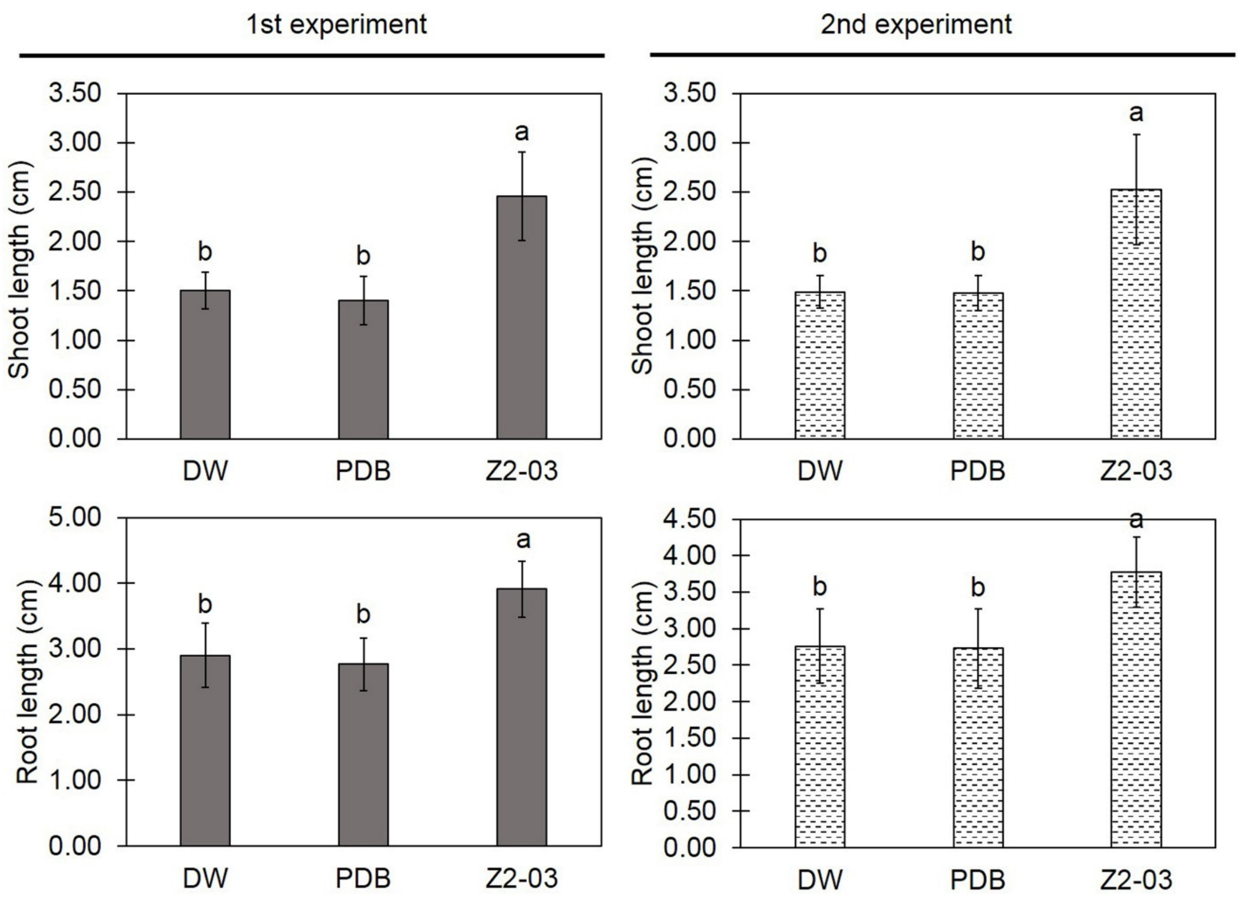

Figure S2. Seed germination, as well as shoot and root length, of "Chor Khing" rice variety assessed using distilled water (DW), potato dextrose agar broth (PDB) and the cell-free culture filtrate of *Trichoderma* isolate Z2-03 (Z2-03) from 1st and 2nd experiment at 5 day post application (dpa). Values are shown as mean ± SD, with letters indicating significant differences among the control and the treatment according to Tukey's test ($p < 0.05$).

2. Increase in Rice Seedling Growth caused by Trichoderma isolate Z2-03

The results show that the shoot and root lengths and the biomass of rice seedlings treated with *Trichoderma* isolate Z2-03 were significantly greater than those of the control ($p < 0.05$). The shoot length of rice seedlings in the control and treatment groups were 12.2 ± 1.22 and 15.30 ± 1.20 cm, respectively, whereas the root lengths of rice seedlings in the control and treatment groups were 8.85 ± 2.01 and 12.74 ± 1.97 cm, respectively (Fig. 3S). The fresh weights of the rice shoots were 0.196 ± 0.006 and 0.286 ± 0.015 mg per five seedlings for the *Trichoderma* isolate Z2-03-treated group and the control group, respectively. The root weights showed the same patterns, and the shoot weights were 0.050 ± 0.003 and 0.131 ± 0.002 mg per five seedlings, respectively (Fig. 3S). The results of the second experiment also indicated that treatment with *Trichoderma* sp. Z2-03 spore suspension resulted in the highest shoot and root length, as well as the highest fresh weight of shoots and roots of rice seedlings.

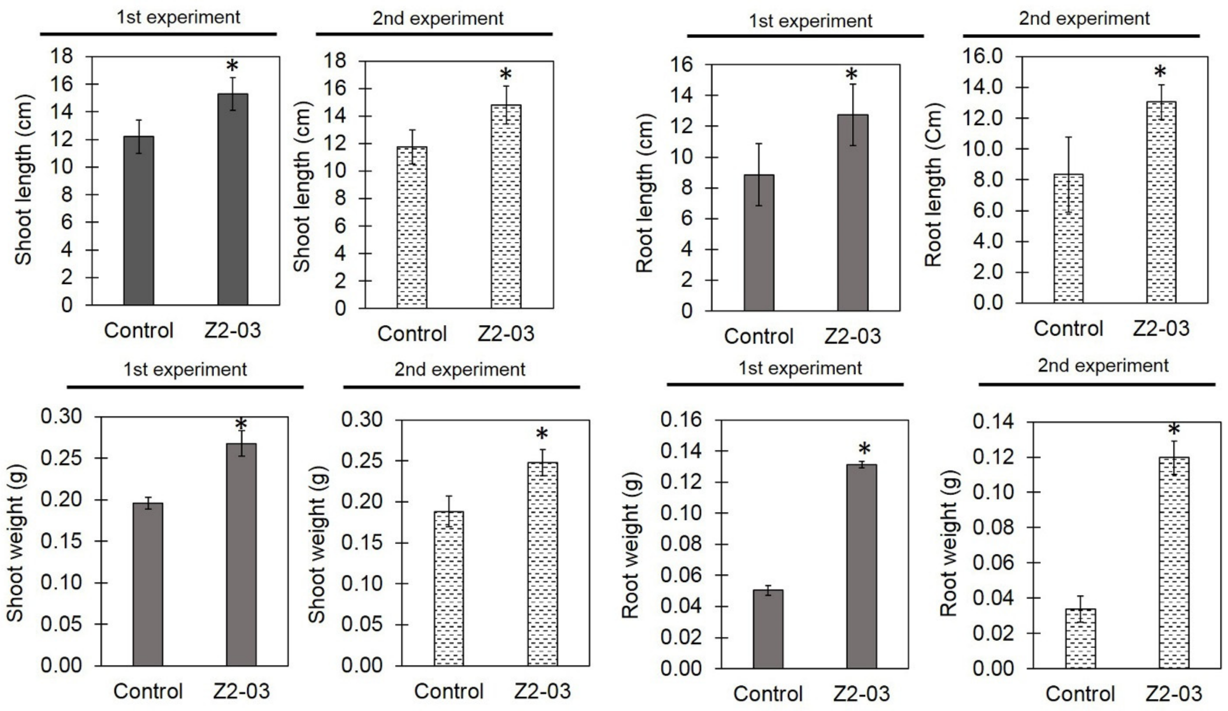

Figure S3. Shoot and root length and biomass of "Chor Khing" rice variety assessed by distilled water (control) and spore suspension of *Trichoderma* isolate Z2-03#from 1st and 2nd experiment. Values are mean ± SD; asterisks indicate significant difference between control and treatment groups according to Student's t-test ($p < 0.05$).

3. Total Chlorophyll Content

The total chlorophyll contents of the *Trichoderma* isolate Z2-03-treated group and the group were measured; the former was 5.093 ± 0.054 mg, which is significantly higher than that of the control, at 1.352 ± 0.001 mg (Fig. 4S). The results of the repeated experiment (experiment 2) also demonstrated that treatment with *Trichoderma* sp. Z2-03 spore suspension resulted in the highest total chlorophyll content (Fig. 4S).

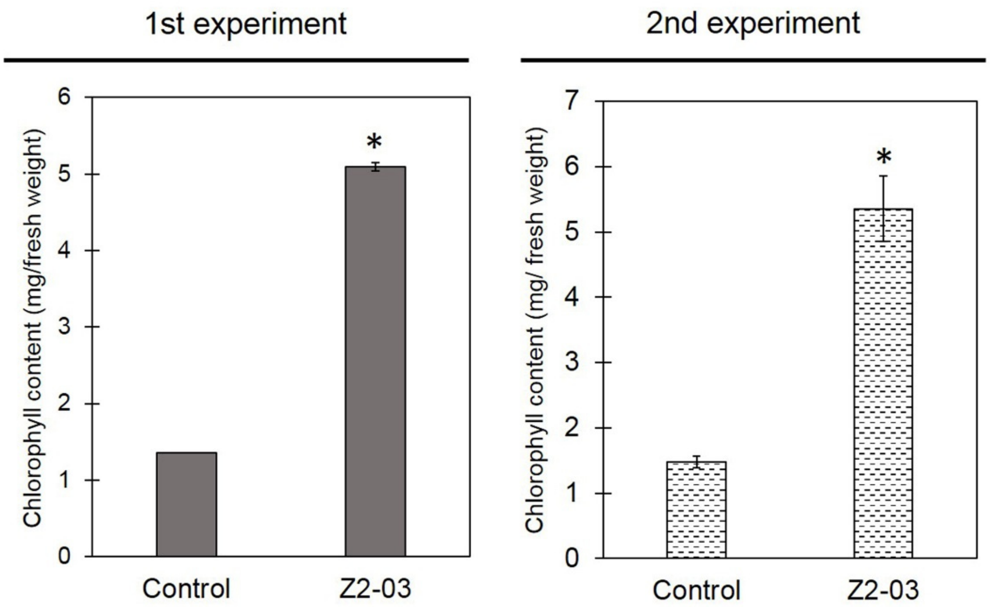

#

Figure S4. Total chlorophyll content of "Chor Khing" rice variety assessed by distilled water (control) and spore suspension of *Trichoderma* isolate Z2-03#from 1st and 2nd experiment. Values are mean ± SD; asterisks indicate significant difference between control and treatment groups according to Student's t-test ($p < 0.05$).

4. Antifungal Ability of Trichoderma sp. Z2-03

At 7 days of incubation, the mycelial radius of *R. solani* on the control plate was 6 cm, which is significantly higher than that on the plate tested with *Trichoderma* isolate Z2-03 (Fig. 5S). The results of the repeated experiment (experiment 2) also exhibited the same trend, with the mycelial radius of *R. solani* in control plates being significantly higher than that on the tested plates (Fig. 5S).

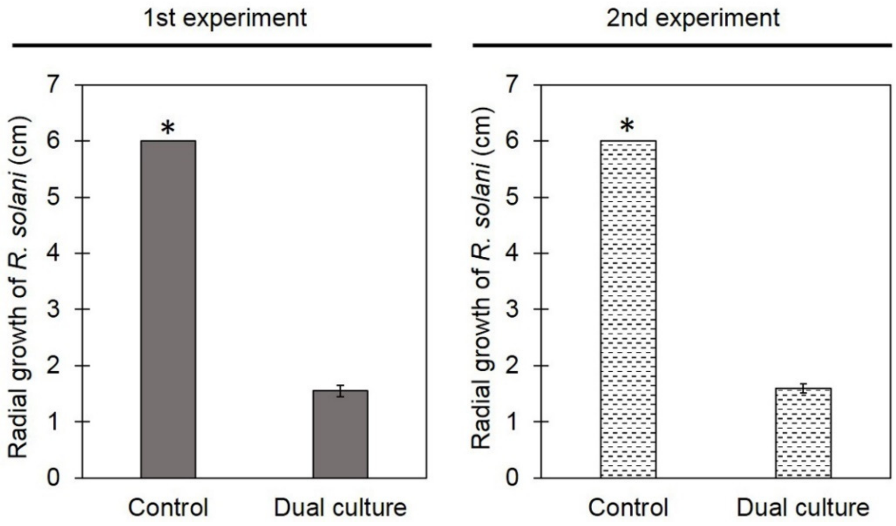

Figure S5. Mycelial radii of *R. solani* in control and dual culture plates of 1st and 2nd experiment. Values are mean ± SD, asterisks indicate significant difference between control and treatment according to Student's t-test ($p < 0.05$).

5. Siderophore Production of Trichoderma zp. Z2-03

Both experiments (1st and 2nd experiments) showed *Trichoderma* sp. Z2-03 produced siderophore, indicated by a color change of the CAS media from blue to yellow from day 5 to day 7 (Fig. 6S).

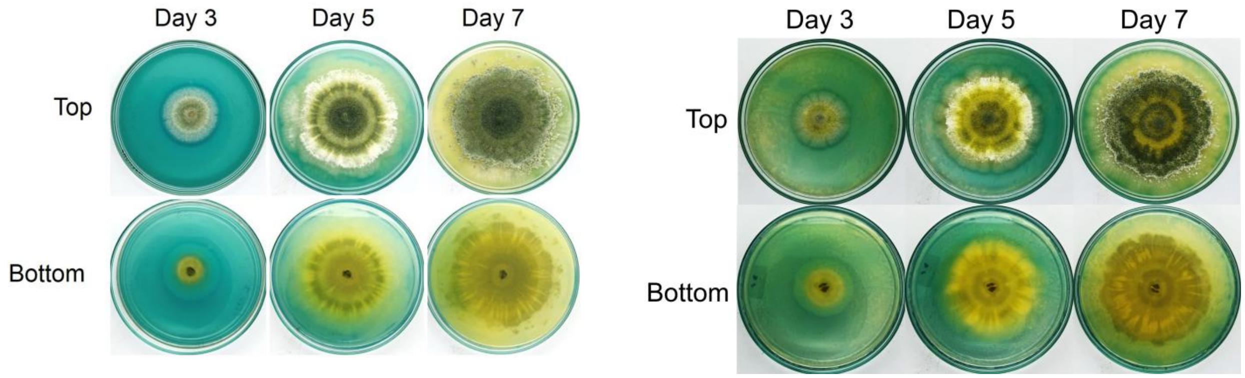

Figure S6. Siderophore production of Trichoderma isolate Z2-03 on CAS medium was monitored at ambient temperature on days 3, 5, and 7 post-incubation. The presence of a yellow clear zone on the CAS medium indicates siderophore production. The results on the left side correspond to the 1st experiment, while those on the right side correspond to the 2nd experiment.

6. *Trichoderma Promote Plant Growth and Reduce Disease Incidence*

After incubation for 30 days, the application of the *Trichoderma* isolate's Z2-03 conidial suspension before inoculation with the *R. solani* treatment (T1) yielded 3.63 tillers, which is significantly higher than the numbers in other treatment (Fig. 7S). Regarding plant height, the application of the *Trichoderma* isolate Z2-03 conidial suspension yielded a height of 63.50 cm, which is significantly greater than the heights measured in other treatment (Fig. 6S). Furthermore, the application of the *Trichoderma* isolate Z2-03 conidial suspension led to a PDI of 67.85%, which is significantly lower than those measured in other treatments (Fig. 7S). The results of the second experiment also demonstrated that the number of tillers and plant height of T1 were significantly higher compared to the other treatments (Fig. 7S). Additionally, the application of the *Trichoderma* isolate Z2-03 conidial suspension resulted in a PDI of 71.43%, which is significantly lower than that observed in the other treatments (Fig. 7S).

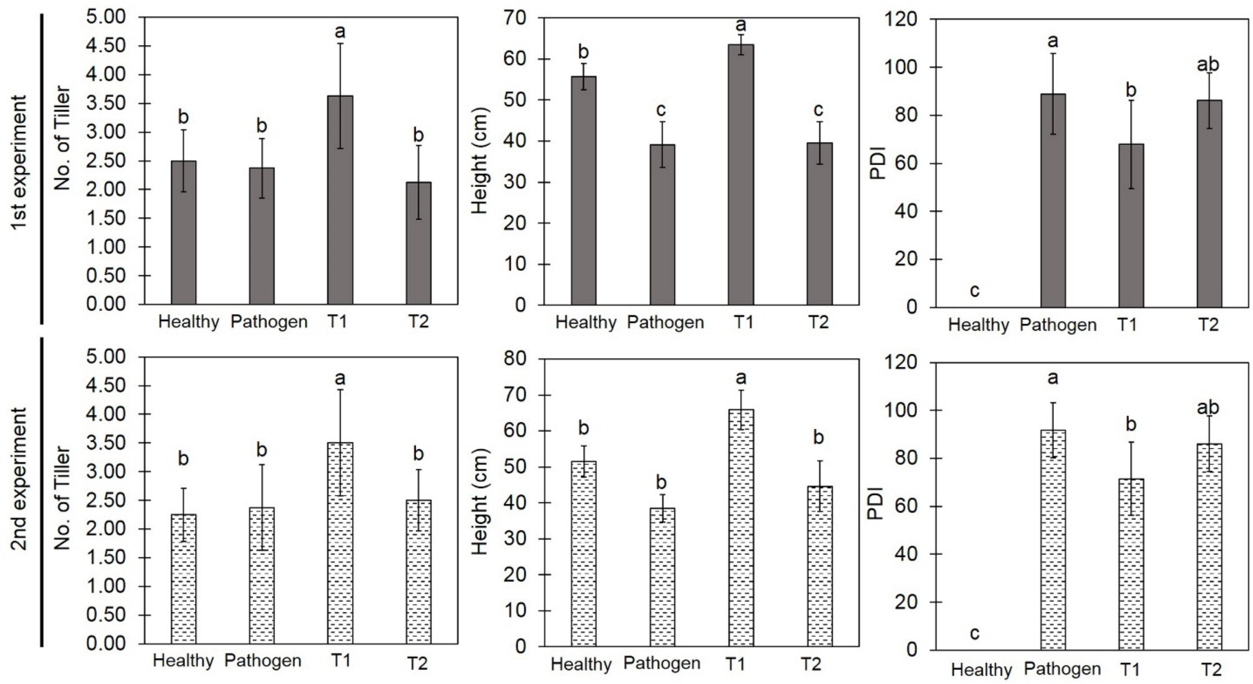

Figure S7. Effect of conidial suspension of *Trichoderma* isolate Z2-03 on number of tillers, plant height and percentage of disease incidence from 1st and 2nd experiments. Values are means ± SD and letters indicate significant difference among treatments according to Tukey's test (p<0.05). T1 indicates the application of *Trichoderma* isolate Z2-03 prior to *Rhizoctonia solani* inoculation, whereas T2 indicates inoculation with *R. solani* prior to *Trichoderma* isolate Z2-03 application.
